# Functional Phenotypic Rescue of *Caenorhabditis elegans* Neuroligin-Deficient Mutants by the Human and Rat *NLGN1* Genes

**DOI:** 10.1371/journal.pone.0039277

**Published:** 2012-06-18

**Authors:** Fernando Calahorro, Manuel Ruiz-Rubio

**Affiliations:** 1 Departamento de Genética, Facultad de Ciencias, Universidad de Córdoba, Córdoba, Spain; 2 Instituto Maimónides de Investigación Biomédica de Córdoba (IMIBIC), Córdoba, Spain; Inserm U869, France

## Abstract

Neuroligins are cell adhesion proteins that interact with neurexins at the synapse. This interaction may contribute to differentiation, plasticity and specificity of synapses. In humans, single mutations in neuroligin encoding genes lead to autism spectrum disorder and/or mental retardation. *Caenorhabditis elegans* mutants deficient in *nlg-1*, an orthologue of human neuroligin genes, have defects in different behaviors. Here we show that the expression of human *NLGN1* or rat *Nlgn1* cDNAs in *C. elegans nlg-1* mutants rescues the fructose osmotic strength avoidance and gentle touch response phenotypes. Two specific point mutations in *NLGN3* and *NLGN4* genes, involved in autistic spectrum disorder, were further characterized in this experimental system. The R451C allele described in NLGN3, was analyzed with both human NLGN1 (R453C) and worm NLG-1 (R437C) proteins, and both were not functional in rescuing the osmotic avoidance behavior and the gentle touch response phenotype. The D396X allele described in NLGN4, which produces a truncated protein, was studied with human NLGN1 (D432X) and they did not rescue any of the behavioral phenotypes analyzed. In addition, RNAi feeding experiments measuring gentle touch response in wild type strain and worms expressing SID-1 in neurons (which increases the response to dsRNA), both fed with bacteria expressing dsRNA for *nlg-1*, provided evidence for a postsynaptic *in vivo* function of neuroligins both in muscle cells and neurons, equivalent to that proposed in mammals. This finding was further confirmed generating transgenic *nlg-1* deficient mutants expressing NLG-1 under pan-neuronal (*nrx-1*) or pan-muscular (*myo-3*) specific promoters. All these results suggest that the nematode could be used as an *in vivo* model for studying particular synaptic mechanisms with proteins orthologues of humans involved in pervasive developmental disorders.

## Introduction

Animal social interactions, learning experiences and behavioural responses to a wide range of environmental stimuli are fundamentally underpinned by the connectivity between nerve cells at synapses. The synapses act in circuits which serve to integrate and compute an extraordinary complex network of inputs. Behavior is the result of the interaction of a set of varying stimuli with an extraordinary complex network of nervous and muscle cells. Several lines of evidence suggest that the alteration of neuron connections during development of the nervous system may constitute the basis of pervasive developmental disorders [1], [2]. Neuroligins are postsynaptic cell adhesion proteins that interact with neurexin at the synapse [3], [4]. Both neuroligins and neurexins were shown to induce synaptogenesis [5]–[7], and it has been proposed that the interactions between different isoforms contribute to differentiation and specificity of synapses [8], [9]. Most mammals have four neuroligin genes (*NLGN1-4*) [10]–[12] while humans have a fifth gene in the Y chromosome (*NLGN4Y*) [13]. All neuroligins share a similar structure with a large extracellular cholinesterase-like domain, a transmembrane region, and a short cytoplasmic tail [14]. In the vertebrate central nervous system, neuroligins are preferentially localized at postsynaptic sites where they assemble as a dimer, linking two subunits through a tightly packed four-helix bundle of two helices from each subunit [15]. The neurexin/neuroligin adhesion system of synapses is highly conserved in the animal kingdom and although gene number and isoforms vary among vertebrates and invertebrates, these adhesion complexes seems to be conserved throughout evolution [16]. In humans it has been shown that single missense and frameshift mutations in neuroligin genes lead to autism and/or mental retardation with complete penetrance [17]–[21].

The *C. elegans nlg-1* gene is orthologous to mammalian neuroligin genes. The protein encoded by *nlg-1* is similar to other neuroligin proteins, because it conserves the structure of the major domains, including the large extracellular cholinesterase-like domain, a type-1 transmembrane protein sequence and an intracellular domain with a PDZ-binding motif at the C-terminal end [22]. The *nlg-1* gene is expressed throughout the *C. elegans* nervous system, including around 20 neurons of the head, 20 neurons in the ventral nerve cord and in the body wall of some muscle cells [22]. As in vertebrates, NLG-1 is located at dendritic postsynaptic sites, although it is also expressed in presynaptic regions of some types of neurons [23]. The gene covers about 6 Kb in the nematode genome, and as many as 24 NLG-1 isoforms have been predicted as consequence of alternative splicing *nlg-1* [22]. The *nlg-1* deficient mutants are defective in sensory behavior affecting various neuronal circuitries [22], [24]. In addition, sensitivity to oxidative agents such as paraquat and copper, as well as intolerance to mercury, was much higher in *nlg-1*-mutants than in wild-type animals [22].

In this study we show that two behaviors impaired in *nlg-1* deficient mutants, fructose osmotic strength avoidance [25] and gentle touch response (identified in this work), are rescued by transgenic expression of cDNA from human *NLGN1* or rat *Nlgn1* genes, demostrating that these mammalian neuroligins are functional in the nematode. Our results suggest that *C*. *elegans* can be used as an *in vivo* model for the study of specific human genes, and open the way for the analysis of molecular mechanisms involved in synaptic activities involved in complex human neurological disorders.

## Results

### Generation of transgenic strain expressing worm NLG-1, human NLGN1 and rat Nlgn1 in neuroligin deficient mutants of *C*. *elegans*

To test whether mammalian neuroligins were able to rescue the behavioral phenotype of neuroligin deficient mutants of *C. elegans*, we generated translational constructs with the *nlg-1* promoter driving the human *NLGN1* cDNA (Kazuza DNA Research Institute, Japan), the rat *Nlgn1*-*EGFP* construct [26], [27] and as a positive control the worm *nlg-1* cDNA (National Institute of Genetics, Mishima, Japan).

A set of transgenic strains were produced by coinjecting the plasmid carrying the human *NLGN-1* or worm *nlg-1* cDNAs under control of the *nlg-1* promoter, together with a plasmid conferring resistance to neomicine as a selection marker which expressed *GFP* under control of the *myo-2* promoter as an additional transformation marker [28]. Other transgenic strains expressing the human NLGN-1 were obtained by coinjecting the plasmid with *NLGN-1* cDNA under the *nlg-1* promoter and a plasmid expressing GFP driven by the promoter of *nrx-1* as a transformation marker [28]. The worm *nrx-1* gene encodes an orthologue of vertebrate neurexins, that is expressed in most cells of the nervous system [29].

Several lines of worm expressing the rat *Nlgn1*-*EGFP* cDNA were obtained by coninjecting a plasmid harboring this cDNA driven by the *nlg-1* gene promoter together with a plasmid conferring resistance to neomycin [28].

In order to select the transformed worms expressing human or rat NLGN1 for phenotypic analysis, transgenic animals were analyzed under the microscope to monitor GFP or EGFP expression. Figure S1 illustrates the expression of GFP and EGFP under the *nrx-1* and *nlg-1* gene promoters respectively. The expression pattern observed in head ganglia and ventral nerve cord was similar to that reported previously for neurexin [29] and neuroligin [22] genes in the worm.

### Human *NLGN1* and rat *Nlgn1* cDNAs rescue the mutant phenotype in *nlg-1* defective mutants of *C*. *elegans* for the response to 4M fructose solution

**Figure 1:**
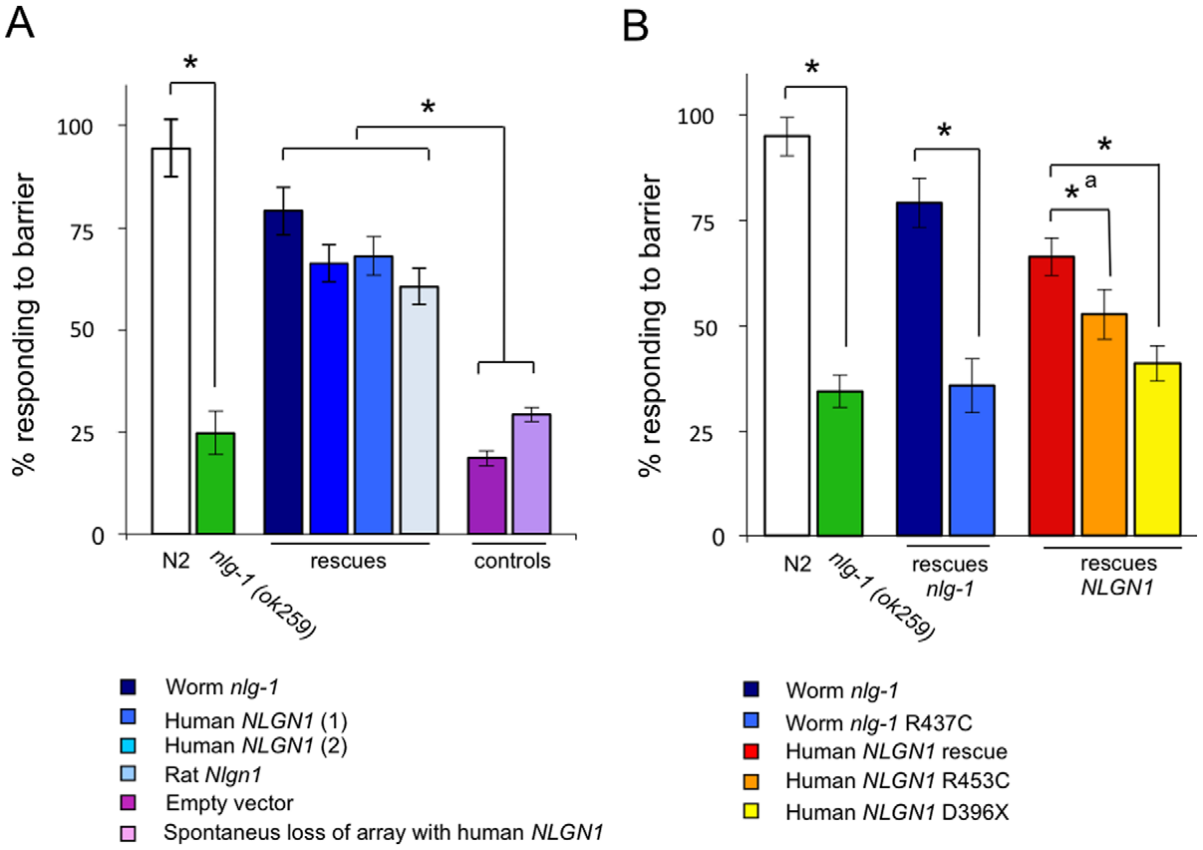
Expression of human *NLGN1* and rat *Nlgn1* cDNAs rescue the osmotic strength response in *nlg-1* defective mutants of *C*. *elegans*. Percentages of osmotic avoidance to 4M fructose solution of wild type N2, *nlg-1*(*ok259*) and different transgenic strains are illustrated. (**A**) Rescues: Worm *nlg-1* correspond to CRR104 transgenic strain (*crrEx4* [pPD95.77 (*Pnlg-1*::*nlg-1*); pDD04Neo^R^ (*Pmyo*-2::*GFP*)]); Human NLGN1 (1) and (2) represent transgenic strains CRR103 (*crrEx3* [pPD95.77 (*Pnlg-1*::*NLGN1*); pDD04Neo^R^ (p*myo-2::GFP*)]) and CRR106 (*crrEx6* [pPD95.77 (*Pnlg-1*::*NLGN1*); *nrx-1*::*GFP*]); Rat *Nlgn1* correspond to transgenic strain CRR109 (*crrEx9* [pPD95.77 (*Pnlg-1::Nlgn1::EGFP*); pBCN24Neo^R^]). Controls are CRR100 transgenic strain containing the pDD04Neo^R^ empty vector, and a strain derived from CRR106 that spontaneously lost the array with *GFP* and human *NLGN1 cDNA*. (**B**) Rescues: Worm *nlg-1* is CRR104 strain as above; Worm *nlg-1* R437C is CRR105 strain (*crrEx5* [pPD95.77 (*Pnlg-1::nlg-1*-R437C); pDD04Neo^R^ (*Pmyo*-2::*GFP*)]); Human NLGN1 is CRR103 as above; Human *NLGN1* R453C is CRR107 strain (*crrEx7* [pPD95.77 (*Pnlg-1::NLGN1*-R453C); pDD04Neo^R^ (*Pmyo*-2::*GFP*)]); Human *NLGN1* D396X is CRR108 (*crrEx8* [pPD95.77 (*Pnlg-1::NLGN1*-D396X); pDD04Neo^R^ (*Pmyo*-2::*GFP*)]). At least three different experiments were carried out with each strain (approximately 50 worms per experiment). In the case of the strain CRR107, six experiments were performed. Error bars indicate the SEM. The asterisk indicates significant differences (p≤0.001) (_*_^a^ indicates P = 0.014) calculated by ANOVA/Fisher's test.

*C. elegans* has developed mechanisms to avoid hyperosmotic environments such as high concentration of salts or sugars [30], [31]. We have previously shown that *nlg-1* deficient mutants are impaired in the capacity to detect a 4M fructose solution [24], [25]. Figure 1A shows that the average percentage of response to the osmotic barrier for Bristol N2 wild type strain was 95±7%. This response is notably much higher than 25±5 % presented by the *nlg-1* deficient strain carrying the allele *ok259*. Figure 1A also shows that transgenic expression in the *nlg-1(ok259)* deficient mutant of cDNAs from *C*. *elegans nlg-1*, human *NLGN1* or rat *Nlgn1*, all driven by the *C*. *elegans nlg-1* promoter, rescued the mutant phenotype. Percentage responses in these transgenic strains were 79±6% for worm *nlg-1* cDNA, 66±5% and 68±5% for human *NLGN1* cDNA (strains CRR103 and CRR106 respectively), and 61±5% for rat *Nlgn1* cDNA (strain CRR109). Two negative controls consisting of a *nlg-1(ok259)* mutant harboring an empty vector (strain CRR100) and a strain derived from CRR106 that spontaneously lost the array containing GFP and the human *NLGN1 cDNA*, presented percentage responses similar to those of the *nlg-1* (*ok259*) mutant (19±2% and 29±2%, respectively, Figure 1A).

The first two mutations involved in autism spectrum disorder identified in genes encoding neuroligins were a C to T transversion in *NLGN3*, which originated an Arg451Cys change, and a T insertion in *NLGN4* that generated an Asp396X stop codon [17]. Arg451 (NLGN3) is conserved both in worm NLG-1 (Arg437 site) and human NLGN1 (Arg453 site) proteins (c). Asp396 of NLGN4 is conserved in human NLGN1 and corresponds to the Asp432 site (Figure S2). Figure 1B shows the percentage responses to 4M fructose barrier of *nlg-1(ok259)* deficient mutant expressing *cDNAs* of the *C*. *elegans nlg-1* allele encoding a protein with Arg437Cys (CRR105 strain), or human *NLGN1* allele encoding proteins with Arg453Cys (CRR107 strain) and Asp432X (CRR108 strain) changes. While the CRR105 strain showed a clear reduced response to the 4M fructose barrier compared to the CRR104 strain (36±6% versus 79±6%), CRR107 strain only show a slight significant difference (P = 0.014) with its control partner CRR103 strain (53±0.06% versus 66±5%). On the other hand the CRR108 transgenic strain, with the NLGN1 (Asp432X) truncated protein has a similar response to the CRR1 *nlg-1(ok259)* strain (41±4% versus 34±4%) (Figure 1B).

### Human *NLGN1* and rat *Nlgn1* cDNAs rescue the mutant phenotype for gentle touch response in *nlg-1* defective mutants of *C*. *elegans*

**Figure 2:**
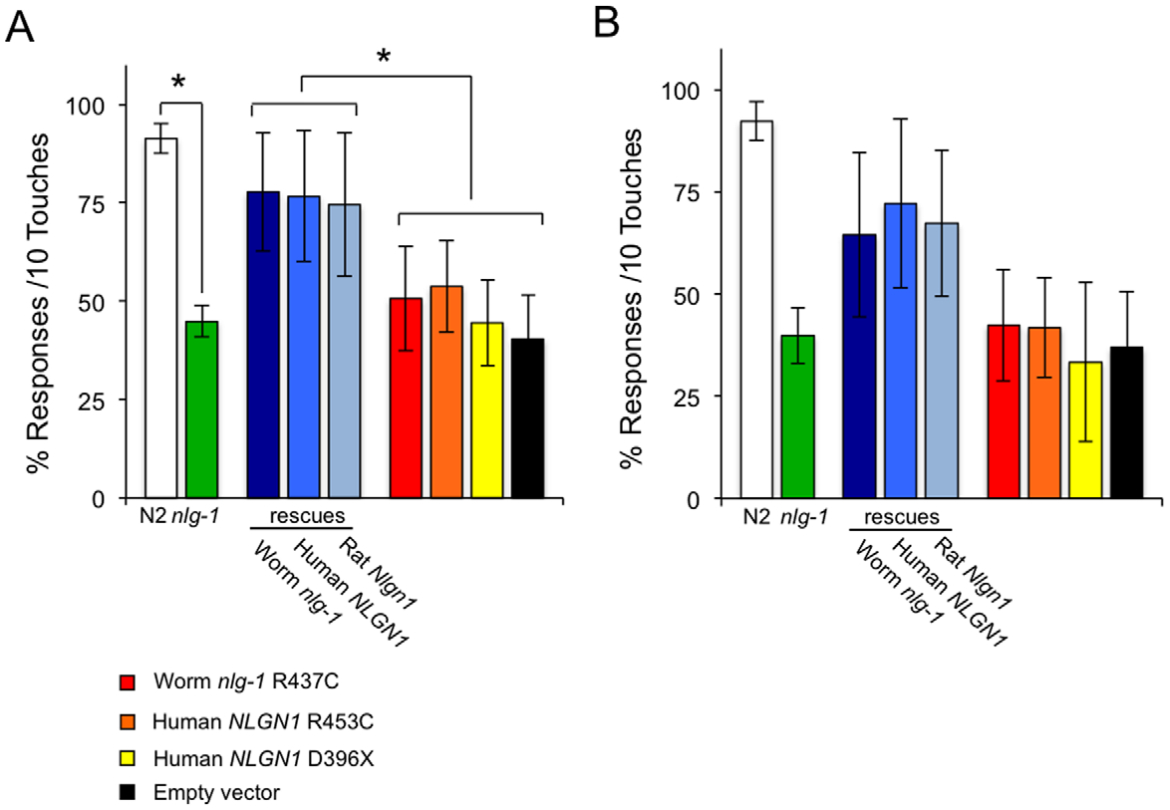
Expression of human *NLGN1* and rat *Nlgn1* cDNAs rescue the wild type phenotype for gentle touch response in *nlg-1* defective mutants of *C*. *elegans*. Data are quantified as percentage of positive response to gentle touch. Animals were touched ten times, alternating anterior and posterior part of the body. The measure was carried out by stroking an eyebrow hair across the body just behind the pharynx for the anterior touch response (**A**), or just before the anus for the posterior touch response (**B**). Rescues: Worm *nlg-1* correspond to CRR104 transgenic strain (*crrEx4* [pPD95.77 (*Pnlg-1*::*nlg-1*); pDD04Neo^R^ (*Pmyo*-2::*GFP*)]); Human NLGN1 represent transgenic strains CRR103 (*crrEx3* [pPD95.77 (*Pnlg-1*::*NLGN1*); pDD04Neo^R^ (p*myo-2::GFP*)]); Rat *Nlgn1* correspond to transgenic strain CRR109 (*crrEx9* [pPD95.77 (*Pnlg-1::Nlgn1::EGFP*); pBCN24Neo^R^]); Worm *nlg-1* R437C is CRR105 strain (*crrEx5* [pPD95.77 (*Pnlg-1::nlg-1*-R437C); pDD04Neo^R^ (*Pmyo*-2::*GFP*)]); Human *NLGN1* R453C is CRR107 strain (*crrEx7* [pPD95.77 (*Pnlg-1::NLGN1*-R453C); pDD04Neo^R^ (*Pmyo*-2::*GFP*)]); Human *NLGN1* D396X is CRR108 (*crrEx8* [pPD95.77 (*Pnlg-1::NLGN1*-D396X); pDD04Neo^R^ (*Pmyo*-2::*GFP*)]). Control is CRR100 a transgenic strain containing the pDD04Neo^R^ “empty vector". At least three different experiments were carried out with each strain (approximately 50 worms per experiment). Error bars indicate the SEM. The asterisk indicates significant differences (p≤0.001) calculated by ANOVA/Fisher's test.

The sense of gentle touch is based on the capability of some sensory cells of *C*. *elegans* to translate mechanical inputs into ionic currents which activate a neural circuit that drives a locomotory response [32]. When the nematode receives a tactile stimulus with an eyebrow hair in the anterior or posterior part of its body, it changes the direction of motion inducing movement back or forward respectively [33]. Figure 2 shows that the neuroligin deficient mutant *nlg-1(ok259)* loses a significant capability of the mechanosensory response in both the anterior and posterior part of the body with respect to the wild type strain. Transgenic expression in the *nlg-1(ok259)* deficient mutant of cDNAs from *C*. *elegans nlg-1*, human *NLGN1* or rat *Nlgn1*, all driven by the *C*. *elegans nlg-1* promoter, rescued the mutant phenotype (Figure 2), whereas expression of *cDNAs* from worm *nlg-1* Arg437Cys or human NLGN1 Arg453Cys alleles did not. The transgenic *nlg-1(ok259)* deficient mutants harboring an empty vector or with a human truncated NLGN1 allele (Asp432X) were used as negative controls.

**Figure 3:**
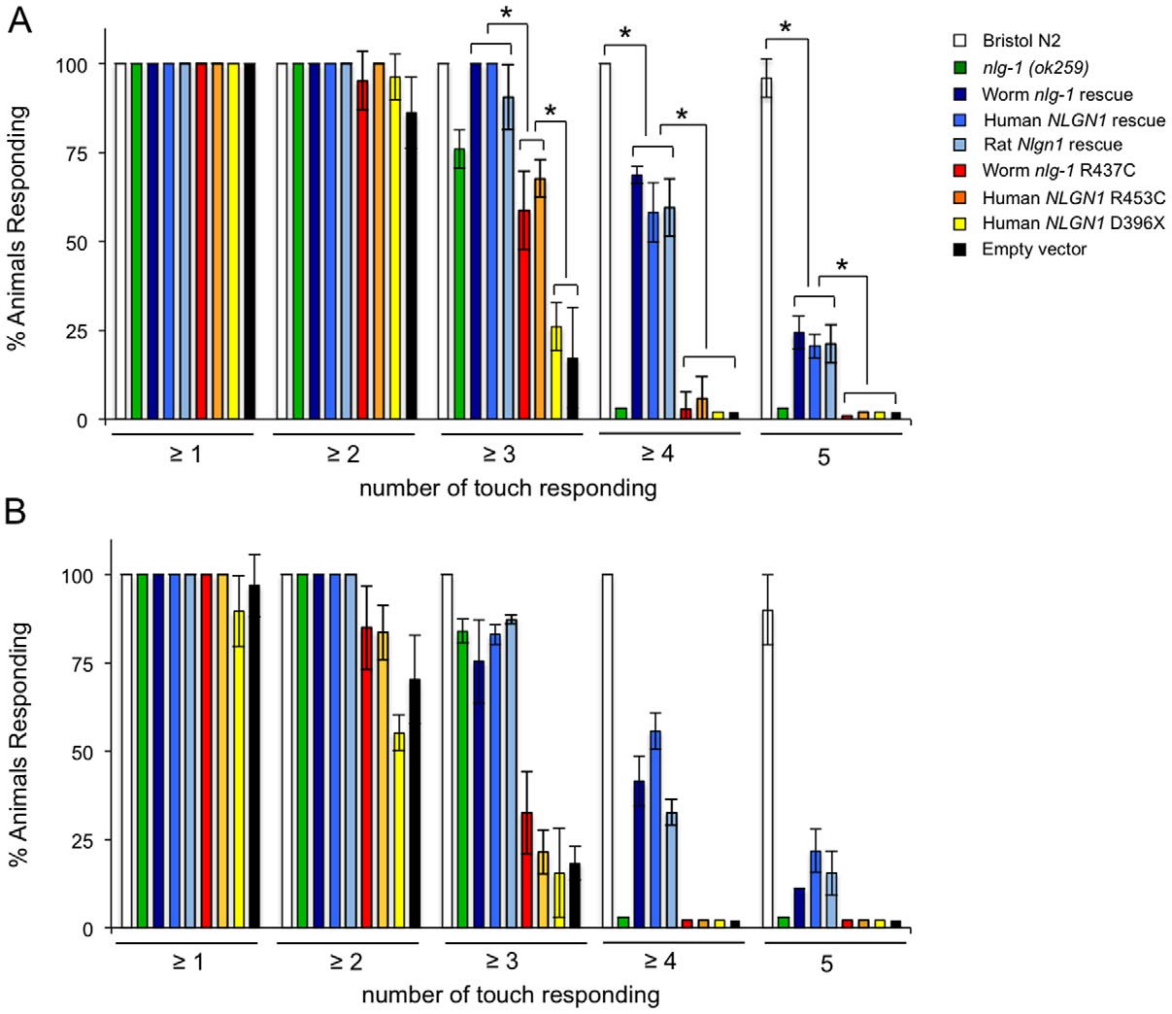
Recovery of the wild type repeated gentle touch response by expression of human *NLGN1* and rat *Nlgn1* cDNAs in *nlg-1* defective mutants of *C*. *elegans*. Data are quantified as percentage of positive responses to ten alternative gentle touches in the anterior (**A**) and posterior (**B**) part of the body (five each). The genotypes of the strains used are shown in the legend of Figure 2. At least three different experiments were carried out with each strain (approximately 50 worms per experiment). Error bars indicate the SEM. The asterisk indicates significant differences (p≤0.001) calculated by ANOVA/Fisher's test.

When *C*. *elegans* is stimulated repeatedly with an eyebrow hair (gentle touch), the stimulus fails to produce a response and the animal become refractory [33]. Figure 3 shows that the wild type strain responds five times consecutively to gentle touch in the anterior and posterior parts of the body, whereas the neuroligin deficient strain *nlg-1(ok259)* fails to respond almost completely to the fourth and fifth touch. Transgenic expression of cDNAs from *C*. *elegans nlg-1*, human *NLGN1* or rat *Nlgn1*, all driven by the *C*. *elegans nlg-1* promoter, in the *nlg-1(ok259)* deficient mutant, partially rescued the mutant phenotype, while the cDNAs from worm *nlg-1* Arg437Cys or human NLGN1 Arg453Cys alleles failed to rescue the response to the fourth and fifth gentle touch (Figure 3). As negative controls, the transgenic *nlg-1(ok259)* deficient mutants harboring an empty vector or with a human truncated NLGN1 allele (Asp432X) were used.

### Specific expression of worm NLG-1 in neuron and muscle cells, and *nlg-1* RNAi bacterial feeding experiments indicate that neuroligin potentiates, but is not essential for, gentle touch response

**Figure 4:**
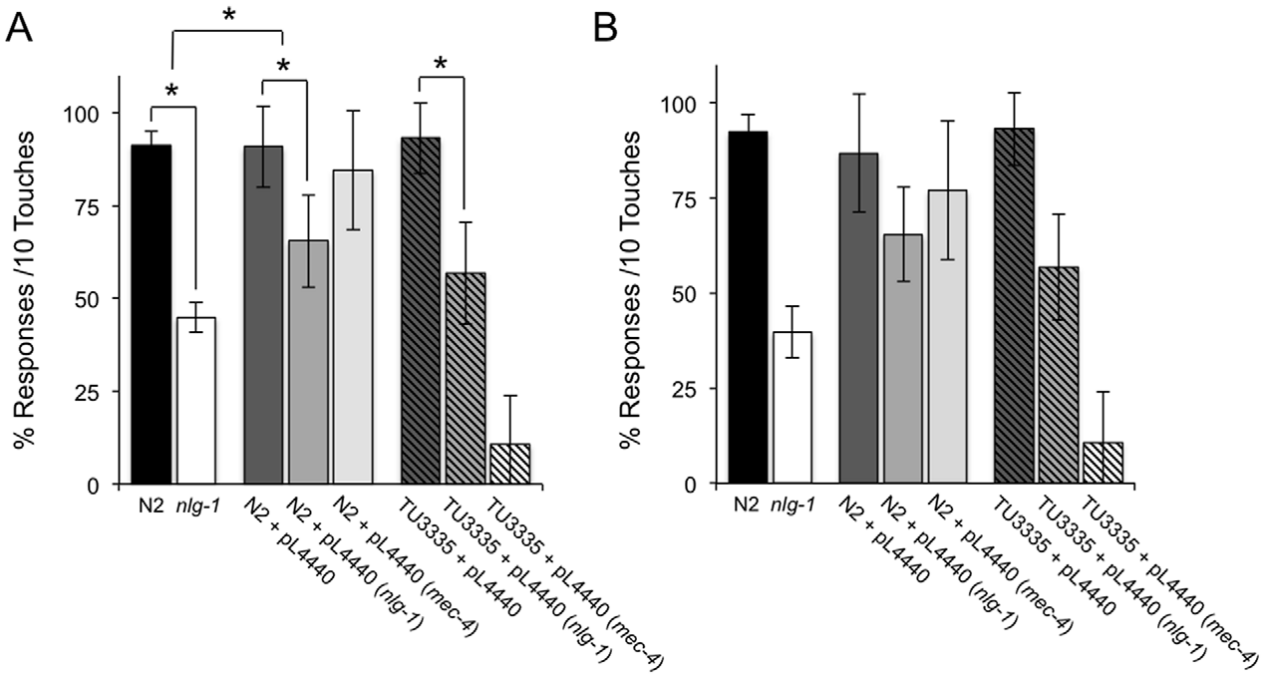
Expression of *sid-1* in neurons does not enhance the response to gentle touch by *nlg-1* RNAi bacterial feeding. Bristol N2 and TU3335 (*P_unc-119_sid-1*) strains were fed with bacteria carrying the pL4440 vector with RNAi targeting the *nlg-1* and *mec-4* genes, or with the empty vector. Quantitative assays for touch sensitivity were carried out in the anterior (**A**) and posterior (**B**) parts of the body. The responses in these knockdown animals were compared with the wild type and *nlg-1* deficient mutants. At least three different experiments were carried out with each strain (approximately 50 worms per experiment). Error bars indicate the SEM. The asterisk indicates significant differences (p≤0.001) calculated by ANOVA/Fisher's test.

Feeding RNAi is efficient in virtually all *C*. *elegans* cells except neurons [34]. We analized the effect of *nlg-1* RNAi bacterial feeding on the gentle touch response phenotype (Figure 4). N2 strain fed with bacteria containing plasmid pL4440 expressing *nlg-1* dsRNA showed a significant decrease in the response to gentle touch respect to control fed with bacteria with empty plasmid, providing evidence for a postsynaptic *in vivo* function of neuroligins in muscle cells of *C*. *elegans*. However, this decrease did not equal the values obtained in *nlg-1* (*ok259*) deficient strain (Figure 4), indicating that neuroligin expressed in neurons also might play a role in gentle touch response. To verify this possibility, we replicate these experiments with the strain TU3335 which specifically express the transmembrane protein SID1 in the nervous cells. SID1 has been proposed to function as a channel for the transport of dsRNA [35], increasing the response to dsRNA delivered by feeding [36]. As shown in Figure 4, RNAi feeding of the TU3335 strain grown on bacteria expressing the *nlg-1* dsRNA fragment produced a similar response to gentle touch that N2 control strain. This result contrast with a control consisting of worms fed with bacteria expressing dsRNA for *mec-4*, a gene expressed exclusively in neurons required for the gentle touch response [32]. These findings suggest that the effect of the deficiency of *nlg-1* in gentle touch depends on the expression of NLG-1 in both muscular and neuronal cells.

**Figure 5:**
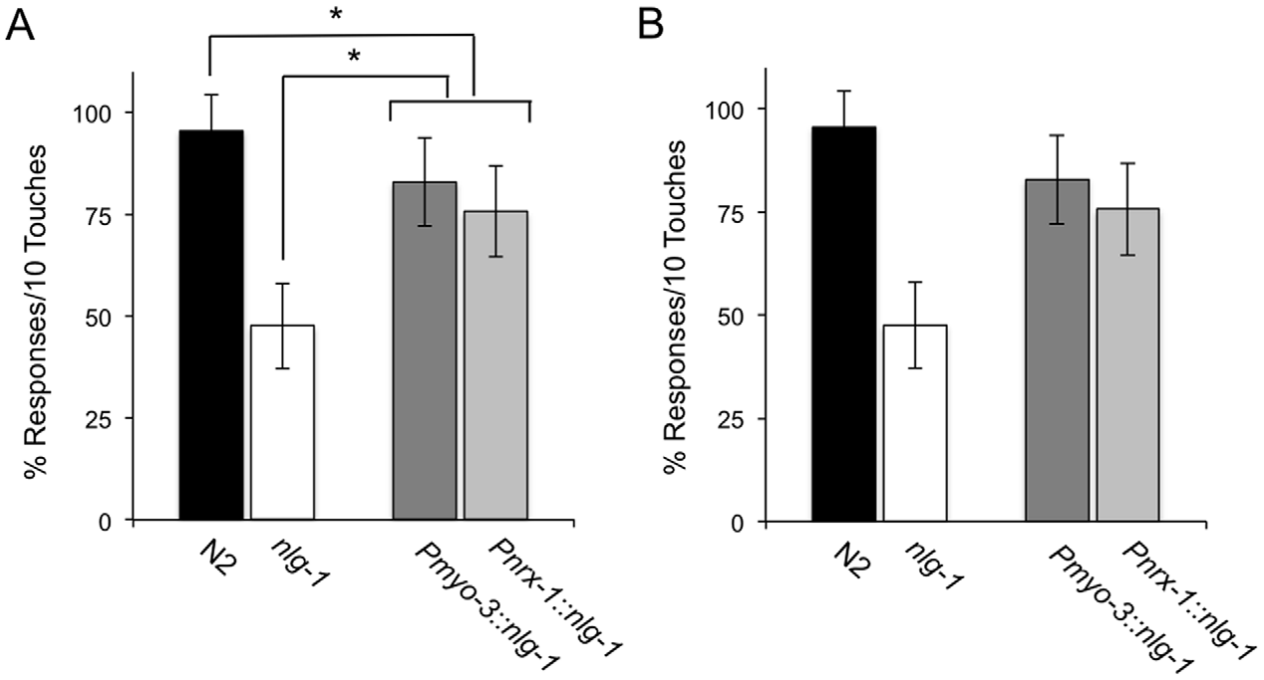
Gentle touch response in transgenic *nlg-1* deficient strains expressing NLG-1 under pan-neuronal (*nrx-1*) or pan-muscular (*myo-3*) specific promoters. Quantitative assays for touch sensitivity were carried out in the anterior (**A**) and posterior (**B**) parts of the body (see legend to Figure 2). The responses in the transgenic animals were compared with the N2 wild type and *nlg-1* (*ok259*) deficient mutants. *Pnrx-1::nlg-1* correspond to strains CRR110 *nlg-1* (*ok259*) *X*; *crrEx10* [pPD95.77 (*Pnrx-1*::*nlg-1*); pDD04Neo_R_ (*Pmyo*-2::*GFP*)], and *Pmyo3::nlg-1* correspond to strain CRR111 *nlg-1* (*ok259*) *X*; *crrEx11*[pPD95.77 (*Pmyo-3*::*nlg-1*); pDD04Neo_R_ (*Pmyo*-2::*GFP*)]. At least three different experiments were carried out with each strain (approximately 50 worms per experiment). Error bars indicate the SEM. The asterisk indicates significant differences (p≤0.001) calculated by ANOVA/Fisher's test.

To further verify the functional role *in vivo* of neuroligin in neurons and/or muscle cells, we generated transgenic *nlg-1* deficient mutants expressing *nlg-1* gene under pan-neuronal (*nrx-1*) or pan-muscular (*myo-3*) specific promoters. Figure 5 shows that both transgenic strains were able to rescue significantly the gentle touch response respect to the *nlg-1* deficient mutant, results that are in agreement with the RNAi bacterial feeding assays; indicating that neuroligin are necessary not only in postsynaptic neuromuscular junction but also in general postsynaptic neuronal wiring. These experiments together with the fact that the *nlg-1* deficient mutant is not completely defective in gentle touch response (Figures 4 and 5) suggest that neuroligin potentiates, but is not essential for, gentle touch response.

## Discussion

Animals are able to detect a wide range of environmental conditions by means of sensory neurons. An unusual feature of these neurons is that they are often polymodal and can respond to distinct types of stimuli. *C. elegans* sensory neurons can express multiple GPCRs in the same nerve cell, allowing the nematode to respond specifically to different environmental conditions using only a few neurons [37]. For this reason, one would expect that mutations in genes encoding proteins involved in a wide range of interaction mechanisms between nerve cells should cause defects in numerous behavioral responses. This is the case of neuroligin, a synapse adhesion molecule conserved widely in the animal kingdom [8]. Thus, *C*. *elegans* mutants deficient in neuroligin were defective in a subset of behaviors and showed hypersensitity to oxidative stress and mercury [22]. The neuroligin deficient mutants were also impaired in the processing of sensory inputs related to the detection of osmotic strength, and failed to consistently detect a 4M fructose solution [24], [25]. Moreover, *nlg-1* deficient mutants are impaired in gentle touch response (Figure 2), and repeated mechanic stimulation with an eyebrow hair led to a reduction in the magnitude of the sensory response in these mutants (Figure 3). These observations indicate that neuroligins are necessary for correct and full overall synapse functionality.

Expression of human *NLGN1* and rat *Nlgn1* isoforms in neuroligin deficient mutants of the nematode showed that both were able to rescue specific behavioral phenotypes such as osmotic avoidance and gentle touch responses. This observation is to some extent expected, given the homology between these proteins. In fact, NLG-1 from *C*. *elegans*, which has an estimated size of 847 amino acids, shows around 28% identity and 44% similarity with the extracellular cholinesterase-like domain of human NLGN1 and rat Nlgn1 proteins (Table S1). The type I transmembrane domain and the intracellular sequence containing the PDZ binding motif, also have significant identity and similarity percentages (Table S1). Furthermore, worm transmembrane proteins have been expressed successfully in human cells [38], and also human transmembrane proteins have been expressed in *C. elegans* [39], indicating that transmembrane domains from proteins of both organisms are functional in either worm or human cell membrane. The homology between mammalian and *C*. *elegans* neuroligins is not only in terms of amino acid sequence, but also in molecular structure. Using the *FlexProt* algorithm [40], the 3D structural alignment of human NLGN1 and *C*. *elegans* NLG-1 proteins showed a analogous spatial functional domain configuration (Figure S3).

It is interesting to note the responses of osmotic avoidance and gentle touch of transgenic strains harboring R437C and R453C mutations in worm and human NLG-1 and NLGN1 proteins, respectively. The R451C change in NLGN3, which has been related to autism spectrum disorder [17], was shown to cause the retention of the protein in the endoplasmic reticulum [41]. The lack of rescue by NLG-1 with the R437C change may have a different cause, since when there is a mutation R451C in NLG-1, two beta-sheets with a greater length between residues 542 and 557 are formed (Figure S4). This could involve a modification in the secondary structure of alpha helix25-beta sheet15-beta harpin-beta sheet16 set, causing a total or partial destabilization of the Ca^2+^ binding site that is necessary for the interaction of neuroligin with neurexins [42]. In the case of human NLGN1, the change R451C did not show any apparent modification in the hypothetical model of protein structure.

The effect of *nlg-1* RNAi bacterial feeding on the gentle touch response phenotype (Figure 4), gives support for a postsynaptic *in vivo* function of neuroligins both in muscle cells and neurons. This idea was further confirm generating transgenic *nlg-1* deficient mutants expressing NLG-1 under pan-neuronal (*nrx-1*) or pan-muscular (*myo-3*) specific promoters (Figure 5). These experiments agree with previous results carried out in a genetic background with a dysfunctional *rrf-3* gene, encoding a putative RNA-directed RNA polymerase, where there is an increase to RNAi sensitivity in most neuron subtypes [43]. It was found that N2 wild type and a *rrf-3* (*pk1436*) strains fed with bacteria expressing a *nlg-1* dsRNA fragment, had a similar behavior with respect to the 4M fructose osmotic avoidance phenotype than the mutation *nlg-1(ok259)* itself [24], [25]; suggesting that there was a contribution from NLG-1 in both neuronal and muscle cell to this behaviour. These overall results suggest that neuroligin potentiates, but is not essential for many of the behavioural phenotypes impaired in *nlg-1* deficient mutants, which explain the viability of these defective strains.

Our results imply that *C*. *elegans* represents a useful *in vivo* genetic model for studying the role of neuroligins in basic mechanistic pathways involved in complex human neurological disorders. The nematode can be used both as a model for studying the behaviour of mutants defective in *nlg-1* and as an organism that allows transgenic and functional expression of mammalian neuroligins. The fact that *C*. *elegans* constitutes a well-characterized system in which neuronal development can be followed simultaneously with molecular and behavioral phenotype analysis, facilitates the analysis of how mutations in neuroligin genes modulate phenotypes in different genetic backgrounds and under different environmental conditions.

## Materials and Methods

### Strains and maintenance

All nematodes were grown and maintained at 20°C under standard conditions [44]. Table 1 shows the worm strains used in this study. *C. elegans* and OP50 E. coli strains were obtained from the *Caenorhabditis* Genetic Center (University of Minnesota, Minneapolis, MN, USA) and the National BioResource Project (Tokyo Women's Medical College, Tokyo, Japan). Extrachromosomal arrays were generated by coinjecting “marker" (0.5 μg/mL) and “rescue" (30–50 μg/mL) plasmids into the germ line of adult hermaphrodites [45].

**Table 1:** *C*. *elegans* strains used in this study

*Strain name*	*Genotype*	*Source*
N2	Wild type, DR subclone of CB original	CGC^a^
VC228	*nlg-1 (ok259) X*	CGC
FX00474	*nlg-1 (tm474) X*	NBP^b^
TU3335	*lin-15B(n744) X; uIs57 [unc-119p::YFP + unc-119p::sid-1 + mec-6p::mec-6]*	CGC
CRR1^c^	*nlg-1 (ok259) X*	This study
CRR2^d^	*nlg-1 (tm474) X*	This study
CRR100	*nlg-1 (ok259) X; crrEx4 [pPD95.77; pDD04 Neo^R^ (Pmyo-2::GFP)]*	This study
CRR103	*nlg-1 (ok259) X; crrEx3* [pPD95.77 (*Pnlg-1::NLGN-1);* pDD04Neo^R^ *(Pmyo-2::GFP)*]	This study
CRR104^e^	nlg-1 (ok259) X; crrEx4 [pPD95.77 *(*Pnlg-*1::nlg-1);* pDD04Neo^R^ *(Pmyo-2::GFP)*]	This study
CRR105	*nlg-1 (ok259) X; crrEx5* [pPD95.77 *(Pnlg-1::nlg-1-R437C);* pDD04Neo^R^ *(Pmyo-2::GFP)*]	This study
CRR106^f^	*nlg-1 (ok259) X; crrEx6* [pPD95.77 *(Pnlg-1::NLGN1); Pnrx-1::GFP*]	This study
CRR107	*nlg-1 (ok259) X; crrEx7* [pPD95.77 *(Pnlg-1::NLGN1-R453C);* pDD04Neo^R^ *(Pmyo-2::GFP)*]	This study
CRR108	*nlg-1 (ok259) X; crrEx8* [pPD95.77 *(Pnlg-1::NLGN1-D396X);* pDD04Neo^R^ *(Pmyo-2::GFP)*]	This study
CRR109^g^	*nlg-1 (ok259) X; crrEx9* [pPD95.77 *(Pnlg-1::Nlgn1::EGFP);* pBCN24Neo^R^]	This study
CRR110	*nlg-1 (ok259) X; crrEx10* [pPD95.77 *(Pnrx-1::nlg-1);* pDD04Neo_R_ *(Pmyo-2::GFP)*]	This study
CRR111	*nlg-1 (ok259) X; crrEx11*[pPD95.77 *(Pmyo-3::nlg-1);* pDD04Neo_R_ *(Pmyo-2::GFP)*]	This study

a *Caenorhabditis* Genetics Center.

b National BioResource Project.

c After outcrossing VC228 strain with N2 at least six times.

d After outcrossing FX00474 strain with N2 at least six times.

e The cDNA *nlg-1* coding region was obtained from clone yk1657a10 from Yuji Kohara, National Institute of Genetics, Mishima, Japan.

f The cDNA human *NLGN1* coding region was obtained from clone KIAA1070 (hj05602), Kazusa DNA Research Institute, Japan.

g Rat *Nlgn-1::EGFP* was a gift from Dr. Thomas Dresbach, Univ. Göttingen, Germany.

### Cloning and transgenic methods

#### cDNA cloning of the *C. elegans nlg-1* gene

Information available at www.wormbase.org, identifies the *C. elegans* gene C40C9.5 as encoding a neuroligin, with three different splices variants named *a*, *b* and *c* respectively. C40C9.5 has been assigned the name *nlg-1*. The genomic sequence of *nlg-1* was used to design the following specific primers, *nlg-1*forward 5′-GCGAATTTG***ggatcc***AACAGGCATG-3′ and *nlg-1*resverse 5′-GGGTGT***gaattc***AAAAACTTGAATTG-3′, to amplified by PCR the coding region corresponding to transcript *b* from the cDNA clone yk1657a10 (from Yuji Kohara, National Institute of Genetics, Mishima, Japan). The promoter region of *nlg-1* was amplified using the P*nlg-1*forward 5′-T***tctaga***CATATTTTTGGGGAGGCTTTC-3′ and P*nlg-1*reverse 5′-GAAGGAGAAGAAGATAAATG***ggatcc***ATGC-3′ from the C40C9 cosmid clon (from Sanger Institute, UK).

#### cDNA cloning of *NLGN1* human gene

*NLGN-1* genomic sequence was used to design the following specific primers: *NLGN1*forward 5′-ATGT***ggatcc***CATGGCACTGCCC-3′ and *NLGN1*resverse 5′-AAAAATAGTTT***gaattc***TCTTATCTGGC-3′. These primers were used to amplify by PCR, the coding region from the cDNA clone KIAA1070 (hj05602) (obtained from Kazusa DNA Research Institute, Japan).

#### cDNA cloning of *Nlgn1* rat gene

cDNA cloning of *Nlgn1-EGFP* was carried out after *BglII*-*BamHI* digestion of pCMV5 vector, that was a gift from Thomas Dresbach [26], [27]. The digestion product was cloned into pPD95.77 vector.

#### Rescue constructions and transgenic methods

Translational constructions were obtained by fusing *nlg-1*, *NLGN1* or *Nlgn1-EGFP* cDNAs with the *nlg-1, nrx-1* or *myo-3* promoters, using the *XbaI*/*BamHI* site in the pDD95.77 vector. The promoter region of *nrx-1* was amplified using the P*nrx-1*forward 5′- ACATTTTTAAACA***tctaga***TTTCTAGG-3′ and P*nrx-1*reverse 5′- CGTTAAGTATGGTCCA***ggatcc***ATCAAAC-3′ from C29A12 cosmid clon (from Sanger Institute, UK). The *myo-3* promoter was obtained from pPD133.54 [46].VC228 *nlg-1* (o*k259*) *X* animals were microinjected with *nlg-1*, *NLGN1* and *Nlgn1-EGFP* “rescue" constructions together with the “marker" plasmids pDD04Neo^R^, pDD04Neo^R^ (*Pmyo-2::GFP*) or pDD95.77 (*Pnrx-1::GFP*).

### Microscopy and imaging

For precise imaging of GFP fluorescence in the nervous system, a Leica DM 5000B microscope was used. Worms were put into 0.5 μL M9 buffer on a thin 2% agarose pad containing an anesthetic (10 mM levamisole), and were monitored with a 40–63X objective magnification. Worms were observed with a DIC optical system, or in the 395 or 615 nm channel for fluorescence. Images were acquired through a Leica IM50 4.0 camera software, and were cropped to size, assembled, and processed using Abode PhotoShop® software.

### Behavioral assays

The behavioral experiments were carried out with two strains deficient in *nlg-1* gene, CRR1 (*ok259*) and CRR2 (*tm474*). The phenotype of strains with *ok259* allele was assessed in more detail, and the strain with *tm474* allele was assayed to confirm that they shared similar phenotypes. In all the behavioral experiments, at least two different lines of each transgenic strain were analyzed to check if they showed similar phenotypes.

#### Osmotic avoidance assay

The assay was performed as was described previously in Calahorro et al. [24]. Briefly, *C*. *elegans* L4 animals of each strain were placed within an annular ring (1 cm diameter) of 4 M fructose solution, on NGM plates. Wild-type animals typically avoid the ring six times in succession. Worms failing to avoid the ring six times consecutively were scored as non-avoiders. The data were expressed as percentage of worms responding to the osmotic barrier.

#### Gentle touch assay

This phenotype was tested by stroking ten times the worm with an eyebrow hair attached to a toothpick, alternating the anterior (just behind the pharynx) and posterior (just before the anus) part of the body. A positive response causes the animal to move backward or forward respectively [33], [47].

### Bacterial feeding RNA interference assay

N2 Bristol and TU3335 *C*. *elegans* strains were use for RNAi experiments (Table 1). HT115 *E.coli* strain (DE3) with plasmid pL4440 carrying *nlg-1* (JA:C40C9.5) and *mec4* (TO1C8.7) gene fragments were provided by Peter Askjaer, Centro Andaluz de Biología del Desarrollo (CABD), Sevilla, Spain, and Julián Cerón, Bellvitge Institute for Biomedical Research (IDIBELL) Barcelona, Spain, respectively. Worms were fed on standard agar plates supplemented with carbenicillin (50 mg/mL-1) and 1 mM IPTG to induce dsRNA production. HT-115 transformed with the empty pL4440, pL4440/*unc-22* (from Peter Askjaer) and pL4440/*gfp* (from Julián Cerón) constructs were also used as controls.

## Supporting Information

Figure S1 **GFP or EGFP fluorescence in transgenic strains expressing worm NLG-1, human NLGN1 or rat Nlgn1 cDNAs in neuroligin deficient mutants of** ***C*** **.** ***elegans*** **.** (**A**): Expression of GFP in neurons of head ganglia (**a**) and ventral nerve cord *(* ***b*** *)* in strain *crrEx6* [pPD95.77 (*Pnlg-1::NLGN1*); *Pnrx-1*::*GFP*]. (**B**): Expression of EGFP in neurons of head ganglia (a) and ventral nerve cord (**b**) in strain (*crrEx9* [pPD95.77 (*Pnlg-1::Nlgn1::EGFP*); pBCN24Neo^R^]). In **A** (**a**) and **B** (**a**), the images correspond to DIC (above), epifluoresce (middle) and merge (below), respectively. In A (**b**) and **B** (**b**) the images correspond to dorsal nerve cord (above) and ventral nerve cord (below). Scale bars are 15 μm. Arrowheads in **A** (**b**) indicate the position of body cell of each motorneuron. Asterisks indicate autofluorescence signal.
(TIF)

Figure S2 **Comparative amino acid sequences of** ***C*** **.** ***elegans*** **and human neuroligins within the acetylcholine-like domain, showing the conserved residues involved in autism spectrum disorder: Arg (R) in NLGN3 and Asp (D) in NLGN4.** The red boxes mark the residues R and D. R is conserved in human and worm neuroligins, and D only in humans. Identical residues are indicated by black boxes and similar residues are shaded in grey. The alignment of protein sequences was performed using the Clustal W method.
(TIF)

Figure S3
**3D structural alignment for** ***C. elegans*** **NLG-1 and human NLGN1 proteins.** Alignment of the carbon skeletons for *C. elegans* NLG-1 protein (blue) and human NLGN1 (red) proteins is shown. RMSD (*Root Mean Square Deviation*) parameter was calculated using *FlexProt* bioinformatics suite [40]. Values for back-bone length, number of flexible regions, match size and matched rigid fragments between both proteins are shown. RMSD  = 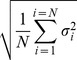; where σ is the distance between N pairs of equivalent Cα.(TIF)

Figure S4 **Hypothetical models of NLG-1 protein of** ***C*** **.** ***elegans*** **.** Wild type NLG-1 protein **(A)** and with the Arg451Cys change **(B)** three-dimensional models, are shown. In **B,** the black arrow indicates the conformational change produced by the R451C mutation, and the white arrow marks the position of α hélix. **C**, shows details of the secondary structure modification in the β sheets 15 and 16 within the 544–557 residues sequence of the protein; thus, the R451C generates longer β sheets between residues 543–557 (black arrow in B). Both models were powered by Swiss-Model Proteomic Serve [48]–[50].
(TIF)

Table S1 Identity and similarity percentage between *C. elegans* NLG-1 and human and rat NLGN1 proteins^a^.
(DOC)
