# Supplementary figures and images for: Functional Phenotypic Rescue of Caenorhabditis elegans Neuroligin-Deficient Mutants by the Human and Rat NLGN1 Genes
Source: PLoS One. 2012 Jun 18;7(6):e39277. doi: 10.1371/journal.pone.0039277 (PMC3377638; doi:10.1371/journal.pone.0039277)

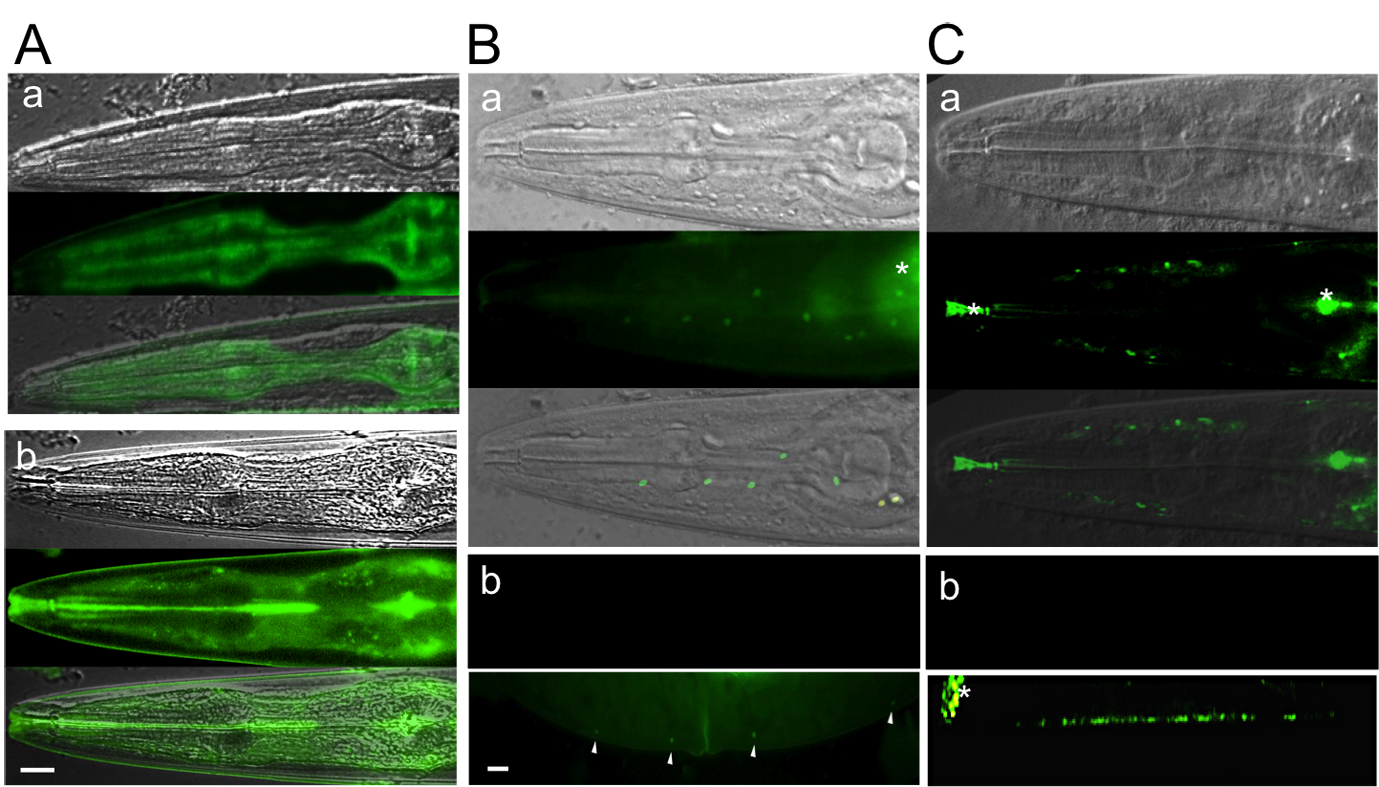

Supplement: Figure S1 — GFP or EGFP fluorescence in transgenic strains expressing worm NLG-1, human NLGN1 or rat Nlgn1 cDNAs in neuroligin deficient mutants of C . elegans . (A): Expression of GFP in neurons of head ganglia (a) and ventral nerve cord ( b ) in strain crrEx6 [pPD95.77 (Pnlg-1::NLGN1); Pnrx-1::GFP]. (B): Expression of EGFP in neurons of head ganglia (a) and ventral nerve cord (b) in strain (crrEx9 [pPD95.77 (Pnlg-1::Nlgn1::EGFP); pBCN24NeoR]). In A (a) and B (a), the images correspond to DIC (above), epifluoresce (middle) and merge (below), respectively. In A (b) and B (b) the images correspond to dorsal nerve cord (above) and ventral nerve cord (below). Scale bars are 15 μm. Arrowheads in A (b) indicate the position of body cell of each motorneuron. Asterisks indicate autofluorescence signal. (TIF) [file pone.0039277.s001.tif]

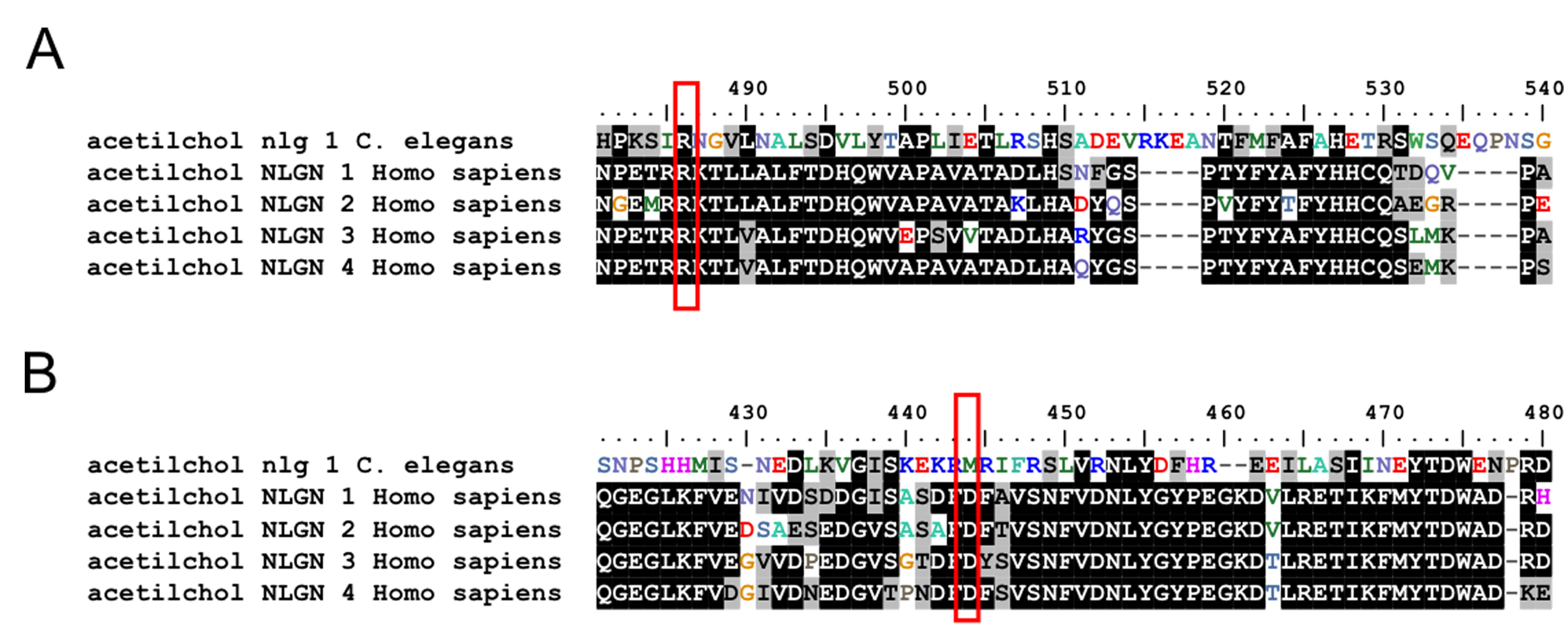

Supplement: Figure S2 — Comparative amino acid sequences of C . elegans and human neuroligins within the acetylcholine-like domain, showing the conserved residues involved in autism spectrum disorder: Arg (R) in NLGN3 and Asp (D) in NLGN4. The red boxes mark the residues R and D. R is conserved in human and worm neuroligins, and D only in humans. Identical residues are indicated by black boxes and similar residues are shaded in grey. The alignment of protein sequences was performed using the Clustal W method. (TIF) [file pone.0039277.s002.tif]

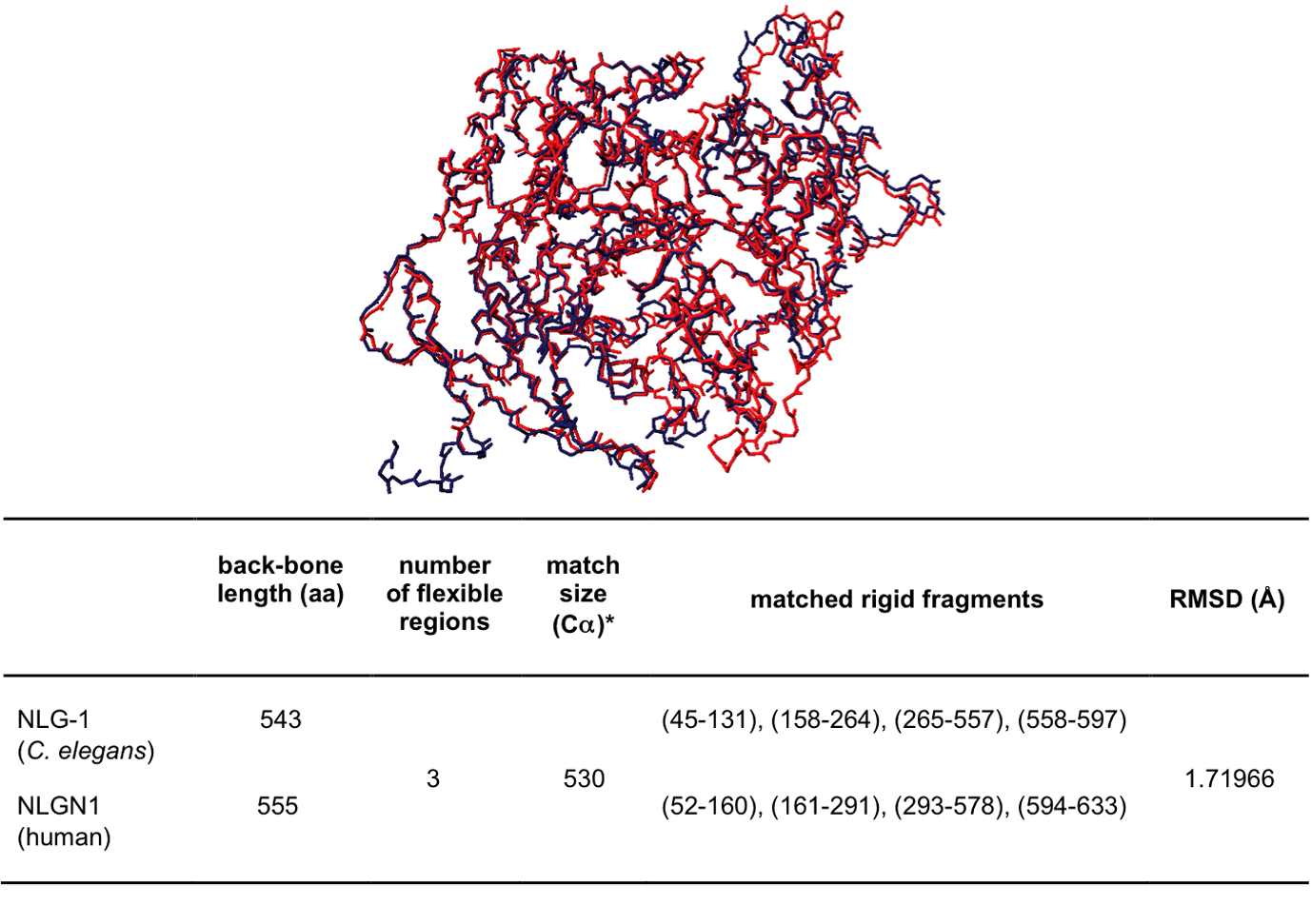

Supplement: Figure S3 — 3D structural alignment for C. elegans NLG-1 and human NLGN1 proteins. Alignment of the carbon skeletons for C. elegans NLG-1 protein (blue) and human NLGN1 (red) proteins is shown. RMSD (Root Mean Square Deviation) parameter was calculated using FlexProt bioinformatics suite [40]. Values for back-bone length, number of flexible regions, match size and matched rigid fragments between both proteins are shown. RMSD = ; where σ is the distance between N pairs of equivalent Cα. (TIF) [file pone.0039277.s003.tif]

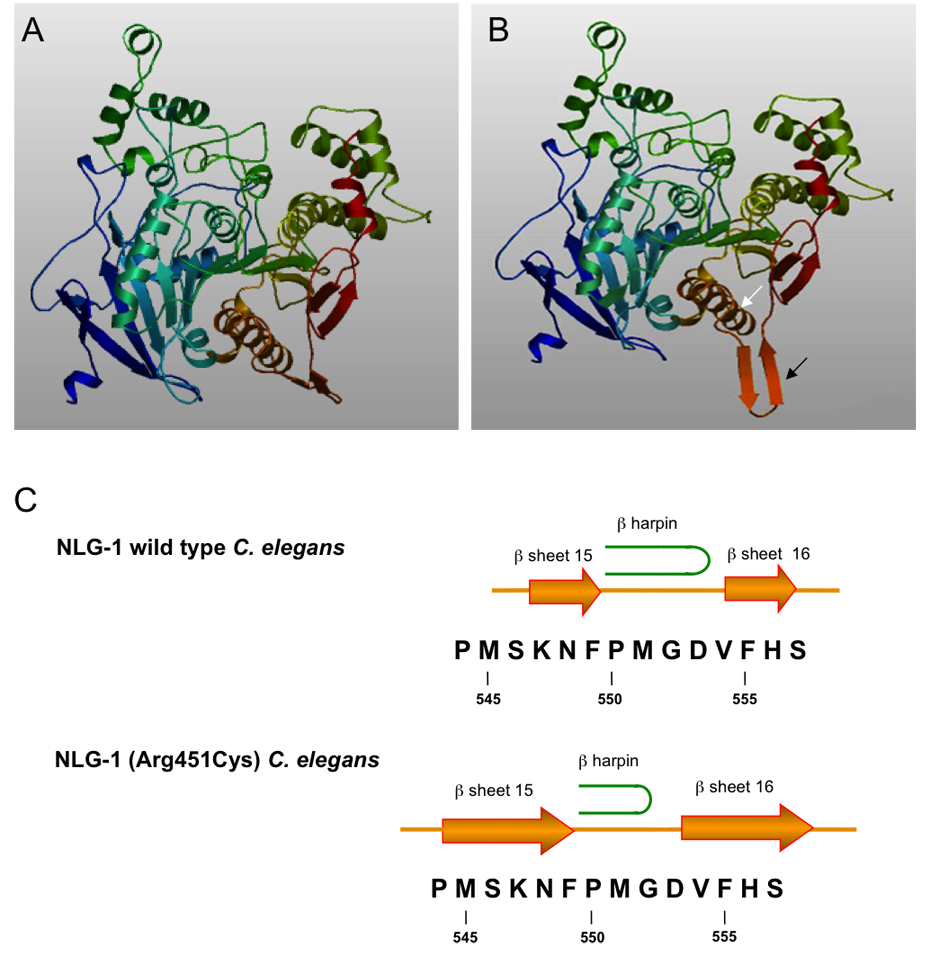

Supplement: Figure S4 — Hypothetical models of NLG-1 protein of C . elegans . Wild type NLG-1 protein (A) and with the Arg451Cys change (B) three-dimensional models, are shown. In B, the black arrow indicates the conformational change produced by the R451C mutation, and the white arrow marks the position of α hélix. C, shows details of the secondary structure modification in the β sheets 15 and 16 within the 544–557 residues sequence of the protein; thus, the R451C generates longer β sheets between residues 543–557 (black arrow in B). Both models were powered by Swiss-Model Proteomic Serve [48]–[50]. (TIF) [file pone.0039277.s004.tif]
