# Supplementary material for: Functional Phenotypic Rescue of Caenorhabditis elegans Neuroligin-Deficient Mutants by the Human and Rat NLGN1 Genes
Source: PLoS One. 2012 Jun 18;7(6):e39277. doi: 10.1371/journal.pone.0039277 (PMC3377638; doi:10.1371/journal.pone.0039277)
Supplement: Table S1 — Identity and similarity percentage between C. elegans NLG-1 and human and rat NLGN1 proteinsa. (DOC) [file pone.0039277.s005.doc]

Table S1. **Identity and similarity percentage between *C. elegans* NLG-1 and human and rat NLGN1 proteins^a^**.

| **Domains** |  | **Human**  **NLGN1** | **Rat**  **Nlgn1** |
| --- | --- | --- | --- |
|  |  |  |  |
| signal peptide | Identity | 4.4 | 6.66 |
|  | Similarity | 22.2 | 8.88 |
| acetylcholinesterase-like | Identity | 28.9 | 27.84 |
|  | Similarity | 44.2 | 43.61 |
| transmembrane | Identity | 22.7 | 22.72 |
|  | Similarity | 40.9 | 40.91 |
| intracellular | Identity | 9.52 | 14.06 |
|  | Similarity | 22.22 | 25.78 |

^a^Pairwise alignment method with BLOSUM62 matrix was used.
